# Development of an integrated and comprehensive clinical trial process management system

**DOI:** 10.1186/s12911-023-02158-8

**Published:** 2023-04-06

**Authors:** Liang Shen, You Zhai, AXiang Pan, Qingwei Zhao, Min Zhou, Jian Liu

**Affiliations:** 1 Department of Information Technology, The First Affiliated Hospital, Zhejiang University School of Medicine, Hangzhou, 310003 China; 2 Research Center for Clinical Pharmacy, Department of Clinical Pharmacy, The First Affiliated Hospital, Zhejiang University School of Medicine, Zhejiang Provincial Key Laboratory for Drug Evaluation and Clinical Research, Hangzhou, 310003 China

**Keywords:** Clinical trial, Clinical trial management system (CTMS), Information technology, Integrated management system, Medical informatics

## Abstract

**Background:**

The process of initiating and completing clinical drug trials in hospital settings is highly complex, with numerous institutional, technical, and record-keeping barriers. In this study, we independently developed an integrated clinical trial management system (CTMS) designed to comprehensively optimize the process management of clinical trials. The CTMS includes system development methods, efficient integration with external business systems, terminology, and standardization protocols, as well as data security and privacy protection.

**Methods:**

The development process proceeded through four stages, including demand analysis and problem collection, system design, system development and testing, system trial operation, and training the whole hospital to operate the system. The integrated CTMS comprises three modules: project approval and review management, clinical trial operations management, and background management modules. These are divided into seven subsystems and 59 internal processes, realizing all the functions necessary to comprehensively perform the process management of clinical trials. Efficient data integration is realized through extract-transform-load, message queue, and remote procedure call services with external systems such as the hospital information system (HIS), laboratory information system (LIS), electronic medical record (EMR), and clinical data repository (CDR). Data security is ensured by adopting corresponding policies for data storage and data access. Privacy protection complies with laws and regulations and de-identifies sensitive patient information.

**Results:**

The integrated CTMS was successfully developed in September 2015 and updated to version 4.2.5 in March 2021. During this period, 1388 study projects were accepted, 43,051 electronic data stored, and 12,144 subjects recruited in the First Affiliated Hospital, Zhejiang University School of Medicine.

**Conclusion:**

The developed integrated CTMS realizes the data management of the entire clinical trials process, providing basic conditions for the efficient, high-quality, and standardized operation of clinical trials.

## Background

Clinical trials of drugs are an important stage in drug research and development and are means to improve the development of medical science and technology [1–5]. Standardizing the management of clinical research is key to simplifying the research process, improving quality, and ensuring the accuracy, reliability, and integrity of the results, thereby shortening the development cycle of new drugs and accelerating the process of drug registration [6]. In China, each hospital has a dedicated clinical trial management department called clinical trial institution (CTI) responsible for the administrative management of clinical trial operation in the hospital. This involves determining the project leader, formulating research plans with sponsors, reviewing by the ethics committee, signing research contracts, screening and enrolling subjects, originating records of trials, receiving drugs, distributing and recovering, processing or reporting adverse events (AE) or serious adverse events (SAE), managing in-hospital quality, accepting supervision and inspection, summary, and filing. The traditional management mode is based on paper documents and involves considerable manual labor, which often leads to untimely information updates, ineffective management, and unverifiable quality. The rapid development of Internet technology has profoundly impacted the mode of clinical trial management. Diverse companies have developed a series of network management systems for clinical trials [7, 8]. Most of these systems have been developed based on the research and development (R&D) requirements of pharmaceutical companies. The clinical trials are managed in a networked manner, which only handles project-related data and excludes the process management links of the clinical trial organization. These commercialized clinical trial data management systems provide comprehensive clinical trial management services, supporting all types of clinical trials, from Phase I to Phase III, from a trial conducted in a single research center to multinational clinical trials; thus, they improve the quality of clinical trials and the efficiency of data management. However, from the perspective of drug clinical trial institutions, this type of clinical trial management system (CTMS) cannot satisfy the practical requirements for real-time and effective management of all clinical trials carried out by these institutions. Hence, a new CTMS has been developed as an institutional management model in China. Its development and application are still in their infancy. The CTMS also faces certain incompatibility issues with the hospital database. It is difficult to achieve data sharing and docking. Moreover, the management method is still relatively primitive. The degree of electronic, networking, and standardization of clinical trials is relatively low, and it is impossible for institutions to effectively manage clinical trials.

In recent years, owing to the increase in clinical trial projects in our hospital, professional and standardized information systems were urgently needed to assist in the management of clinical trials throughout the hospital. At present, the need for a centralized clinical trial project management platform reliant on the clinical data system of the hospital itself is intensifying. Based on the local area network (LAN) security architecture of our hospital, we have constructed a CTMS, which organically integrates the clinical trial organization management office, ethics committee, clinical trial center pharmacy, and clinical professional departments. The management system covers the entire process of clinical trials, including trial project establishment, ethical review, signing of agreements, trial implementation, trial conclusion, sponsor management, subject management, follow-up management, and centralized management of pharmacies supporting medication trials. The personnel involved include clinical departments, institutional management offices, central pharmacies, and ethics committees. With the help of the information system, a large number of personnel and complicated work processes are organically integrated to realize information sharing and collaborative work. With the help of this CTMS, the implementation of clinical trials can be standardized and the entire process can be traceable. As a site of clinical trials, hospital management involves multi-party collaboration, as well as study project management, subject management, investigational product management, quality control, financial management, and other complex business processes. Hospitals must develop dedicated systems to assist the entire process management of clinical trials to ensure their efficient and high-quality operation. The main objectives of this study are as follows. We aim (1) to develop an integrated CTMS as a dedicated database for clinical trials; (2) to achieve efficient data integration of CTMS with hospital business systems (hospital information system (HIS), laboratory information system (LIS), clinical data repository (CDR), picture archiving and communication systems (PACS), and electronic medical record (EMR), etc.); (3) to facilitate standardization and consistency of terminology in the development of database models and business processes; and (4) to ensure the safety and security of clinical data as well as protect patient privacy to comply with relevant regulations.

## Methods

### Overview

The First Affiliated Hospital, Zhejiang University School of Medicine (FAHZU) has six campuses with approximately 5,000 beds. In 2020, the institution recorded over 4.2 million outpatient and emergency visits, and 236,100 discharges. As one of the pioneering and earliest-founded National Drug CTIs in China, the first batch of clinical pharmacology bases under the Ministry of Health was established in 1998. As a first-class integrated service platform for clinical research in China, it operates 24 specialized groups and depends on an internationally recognized and independent ethics examination system, and has undertaken more than 2000 items of foreign and domestic clinical trials since its establishment, including both Phase I–IV drugs and medical devices.

### System development process

The development of an integrated CTMS began in March 2014, and after 18 months, the first version of the CTMS was launched in FAHZU in September 2015. The complete system development process was carried out in four stages, including two months of requirement analysis and problem collection, four months of system design, six months of system development and testing, and six months of system operation training and pilot runs. Based on the feedback from the use of the first version of CTMS and the continuous in-depth exploration of the business, the system’s functionality and user experience continue to be iteratively updated. By March 2021, the updated version 4.2.5 was launched. Through the construction of an integrated CTMS, the entire process of clinical trial data management is realized, which provides the basic conditions for the efficient, high-quality, and standardized operation of clinical trials in the hospital.

#### First stage

The collection and analysis of CTMS requirements defined the existing problem set to be solved. This stage is the most critical and determines the final goal direction of the system. The collection of requirements started in March 2014, and the participants included the CTI office director, CTI office secretary, ethics committee (EC) members, EC secretary, principal investigator (PI), sub-investigator, study nurse, clinical research associate (CRA), clinical research coordinator (CRC), investigational product custodians, financial officer, quality control expert, statisticians, data manager, pharmacovigilance associate, and information technology expert. After two months of formal and informal interviews, as well as an analysis of the collection requirements, the design goal of CTMS was determined. The overall goal of the CTMS is to realize the entire process of clinical trial management, including study project approval, ethical review, subject recruitment, subject management, investigational product management, financial management, and quality management. Table 1 lists the functional requirements for realizing the entire process management of clinical trials in hospitals based on seven dimensions. These requirements were used to construct the integrated CTMS.

**Table 1:** List of functional requirements for realizing the entire process management workflow of clinical trials in hospitals

No	Requirement class	Requirement description
1	Study Project Approval Management	The application and review of clinical trials should adopt process automation management, including process customization configuration, remote submission of project application, uploading of project materials, automatic generation of to-do tasks, timely message transmission, material annotation, and other supporting functions
2	Ethical Review Management	Define ethics committee review process and application contents (new protocols, protocol amendments, etc.)
3	Subject Management	Define the subject management model to ensure that the subjects complete the visit content of each cycle in strict accordance with the research plan
4	Investigational Product Management	The investigational product should adopt the central pharmacy model to achieve closed-loop management, including receiving, warehousing, distributing, recycling, returning, disposal, and early warning
5	Financial Management	Clinical trial finance requires independent accounting and management; the subjects’ diagnosis and treatment processes can be exempted from payment, and the system should automatically record costs to achieve direct settlement between the hospital and the sponsor
6	Quality Management	Define the elements and content of quality management, and the quality control of related data that must be collected during the operation of the system
7	Privilege Management	Involves multi-role collaboration, defining permissions and data access rules for different roles

#### Second stage

**Fig. 1:**
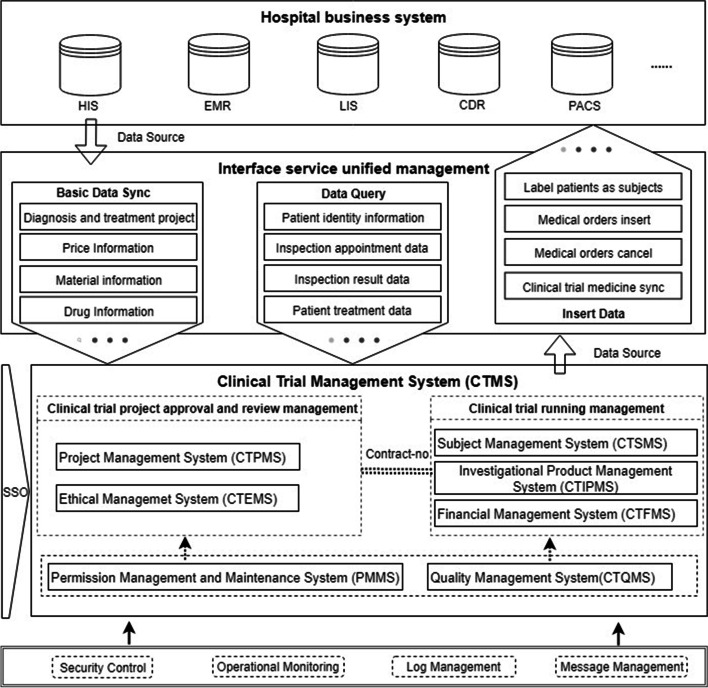
System architecture for the clinical trial management system (CTMS). HIS: hospital information system; EMR: electronic medical record; LIS: laboratory information system; CDR: clinical data repository; PACS: picture archiving and communication systems

In the second stage, the CTMS design process included the definition of the system architecture and data interchange mechanisms, selection of storage media, database modeling and standardization, user interface (UI) and user experience (UX) design, and development of security and privacy policies based on analyzing and categorizing the requirements collected in the first phase, referring to Good Clinical Practice (GCP) and the guidance documents of the National Medical Products Administration (NMPA). The functions and data required by each participant in the clinical trial are determined. Figure 1 shows the system architecture of the CTMS, which is divided into seven subsystems to meet the corresponding requirements of the first stage. Seven dimensions were put forward, including the clinical trial project management system (CTPMS), clinical trial ethical management system (CTEMS), clinical trial subject management system (CTSMS), clinical trial investigational product management system (CTIPMS), clinical trial financial management system (CTFMS), clinical trial quality management system (CTQMS), and permission management and maintenance system (PMMS). These systems include the functions of the whole-process management of clinical trials. The unified control of permissions is realized through single sign-on (SSO) between the systems. To ensure a more efficient operation of the CTMS, it is necessary to focus on the data integration mode with the hospital clinical business system (HIS, LIS, EMR, CDR, etc.).The unified management of interface services is defined as shown in Fig. 1 to complete data integration, which primarily involves three types of data service functions: (1) basic data synchronization, which serves as the basis for system operation; (2) real-time data query to meet the needs of subject information retrieval and clinical trial data monitoring; and (3) diagnosis and treatment data generated by the CTMS, which are transmitted to the clinical business system of the hospital to meet the continuity requirement of diagnosis and treatment of the subjects. Database modeling and standardization are performed with reference to the Clinical Data Interchange Standards Consortium (CDISC) to obtain a standard vocabulary to ensure the integrity of the data model; in terms of system security and subject privacy protection, security protection strategies and development specifications were formulated with reference to the Health Insurance Portability and Accountability Act (HIPAA) and China’s privacy protection laws. In addition, the CTMS implements fine-grained isolation and verification of permissions and records all events in a log to ensure the traceability of the data.

#### Third stage

The third stage focused on the development and testing of the CTMS. This was divided into two steps according to the definition of the design stage. The first step realizes clinical trial approval and review management, and the developed subsystems include CTPMS, CTEMS, and PMMS, which focus on the process management of the study project application and review stage, and do not involve data integration with external systems. The second step realizes clinical trial operational management. The developed subsystems include CTSMS, CTIPMS, CTFMS, and CTQMS, which complete internal function development and data integration with external systems, and realize the entire process of data management. The CTMS was developed based on the distributed architecture of the Java programming language, and its database is Oracle Database 11 g Release 2 on a server running the Linux CentOS 7.6 operating system; data integration with external systems is managed through an interface services unified management platform. The system adopts browser/server architecture and supports current mainstream browsers, such as Google Chrome, Internet Explorer, and Firefox Browser for encrypted access via hypertext transfer protocol secure (HTTPS). System testing mainly includes verification of functional effectiveness, data security, system reliability, and the efficiency of data integration with external systems.

#### Fourth stage

This stage involved CTMS deployment and system operation training. In November 2014, CTPMS, CTEMS, and PMMS were officially launched, realizing clinical trial approval and review management in FAHZU. In March 2015, the development of CTSMS, CTMMS, CTFMS, and CTQMS was completed. Considering that the clinical trial operational management involves data integrity verification and data integration with external systems, a new pilot run phase was needed. By selecting a certain number of clinical trials in Phases I–IV, the operating results were used to verify the compatibility of the system with different types of clinical trials. The CTMS pilot run system first selects Phase I clinical trials and runs a total of 20 projects. Through continuous updating of functions during operation, it can fully meet the needs of Phase I clinical trial operational management. Subsequently, it selects clinical trials in Phases II–IV. A total of 10 projects have been run, and the functions have been stable and meet the requirements of various types of clinical trials. However, training hospital staff in the use of CTMS is critical. Through a variety of methods including face-to-face training and offline data learning, all staff related to clinical trials have been trained. The first version of CTMS was fully launched in FAHZU in September 2015.

## Results

FAHZU is the first hospital in China to independently develop an integrated CTMS; the system has been successfully implemented in the hospital since September 2015. As of March 2021, the hospital now runs an updated version of CTMS known as V4.2.5. The data management of the entire process of clinical trials from project approval and review management to operational management has been fully realized. The FAHZU CTMS operates independently by design as a fully functional system at the application level (not as a component of the HIS), establishes a dedicated clinical trial database at the data level, and completes data integration with external systems through a unified interface system, to achieve process continuity and data integrity. Through the construction of an integrated CTMS, the flow of multi-party collaboration tasks is optimized, and non-research matters such as finance and data processing are simplified, to effectively improve the efficiency and quality of clinical trials. The results shown below are based on the latest stable version of FAHZU CTMS, V4.2.5.

### System categories and features for clinical trial

The first level involves clinical trial project approval and review management, and its functions cover the rationality review of clinical trial study projects and related affairs management by CTI and EC. Table 2 lists the categories and features of clinical trial approval and review management, and the services provided to users through CTPMS and CTEMS. Clinical trial project approval and review management are based on multi-role collaboration, focusing on improving efficiency as the core and supporting remote project application, full electronic project approval, review of process approval documents online, aggregation of reviews into review comments, and timely generation of tasks and notification. The main features of CTPMS include project approval management, to-do task list, project list, document management, contract management, initial meeting management, investigator management, and CRC management. Through the organic combination of these features, the CTI realizes the project approval review and daily management of study projects. The main features of CTEMS include EC management, ethics review management, and ethics conference management, which manages the continuous ethics review of study projects by the EC, including initial protocol review, protocol amendment review, SAE report and review, and violation/deviation protocol review.

**Table 2:** Categories and features for project approval and review management of clinical trials

Category	Features	Description
Study project management	Project approval management	CTI defines the application and review procedures for study projects according to the standard operation procedure (SOP)
	To-do tasks list	Lists the tasks that the user needs to complete, which are automatically generated by the system according to processes and user roles
	Project list	Project list contains basic information about the study, controlling different viewing scopes for different roles
	Document management	Electronic management of documents, batch uploading, online review, and suggestion feedback
	Contract management	Manages the content and budget of the contract, and supervises the execution of the contract
	Initial meeting management	Records the meeting contents and participants of the initial meeting
	Investigator management	Manages investigators’ Good Clinical Practice (GCP) education, resume, etc
	Clinical research coordinator (CRC) management	Manages CRC personnel information, recruitment process, and workload reporting and review
Ethics management	Ethics committee (EC)	Manages the organizational structure of the EC
	Ethics review management	Manages the ethics review process, including ethical review application, formal review, study assessment, ethics conference review, approval letter generation, etc
	Ethics conference management	Manages the project review agenda, meeting attendance, voting, meeting minutes, project review results, etc

The second level comprises clinical trial operational management, which is a complex and continuous management process. Table 3 lists the categories and features of clinical trial operational management; the system provides services to users through the CTSMS, CTIPMS, CTFMS, and CTQMS. CTSMS is mainly composed of three stages: operational management pre-configuration, subject recruitment, and subject visit management. The features included in the operational management pre-configuration stage include study participant assignments, protocol configuration, electronic case report form (e-CRF) design, rule configuration, and global control over the access rights and visitation rules of subjects under the study project. The main features of the subject recruitment stage of the system include subject recruitment, subject violation verification (such as determining whether the subject is participating in another clinical trial and inputting incorrect information of subject), subject lists, and subject global labeling, to realize the registration of subjects under the corresponding study project, status labeling, and visualization. There are three main events in the subject visit management stage, which are described as follows: (1) Obtain the visit content information in the corresponding study cycle according to the study plan and turn it into a to-do list. The visit content includes subject screening, inspection/examination issuance, prescription issuance, treatment, randomization of subjects, e-CRF filling, and AE/SAE reporting. (2) Enter operational status changes of subjects including switching protocols, admission, or discharge, entering the next visit stage, and status of subject updates (dropping out, withdrawal, failure to follow up, etc.). (3) Complete data queries of subjects, including outpatient/inpatient medical records, inspection/examination results queries, subject fee queries, and e-CRF data filling. CTIPMS adopts the central management model, which is managed by qualified personnel designated by the CTI. Through the organic combination of stock management, prescription management, label management, and intelligent detection, closed-loop management of the entire process of trial investigational products is realized. CTFMS primarily combines the financial characteristics of clinical trials and the relevant requirements of the hospital’s financial management, including payment and allocation, budget management, expenditure management, workload statistics, and project funding amounts to achieve orderly financial management based on greatly reducing researchers’ time consumption. Similarly, CTQMS mainly includes regular quality control data report generation and reporting, as well as dynamic quality control based on collected data.

**Table 3:** Categories and features for operational management of clinical trials

Category	Features	Description
Subject management	Study participants assignment	Assigns study project participants (such as investigators, study nurses, clinical research coordinator (CRC), and clinical research associate (CRA)) and sets corresponding permissions
	Protocol configuration	The protocol is mapped to computational executable events; the study plan can be automatically generated according to the visit cycle
	Rule configuration	Configures rules required for subject management (such as inclusion and exclusion criteria, various number generation rules, and drug randomization methods)
	Running projects list	Contains responsible or participating running clinical trial projects, with access management
	Subject list	Contains a list of subjects recruited for this study project, with different colors to distinguish the status of subjects
	Subject recruitment	Subjects’ information can be linked by searching the hospital’s patient database and alerted if they are enrolled in other clinical trials
	Subject study process management	Manages the entire process of subjects from recruitment to the end of the visit, performs the study content required in the corresponding visit cycle (such as screening, randomization, inspection, and investigational product), and can query all the data required for clinical trials
	Study progress statistics	Generates statistics based on the distribution of subjects in different dimensions, including recruitment date, visit cycle, and different states (drop out, withdraw, completion, etc.)
Investigational product management	Stock management	Manages the inbound, storage, outbound, and refund of investigational product; the inventory quantity can be classified and counted by dictionary, batch, and random code
	Prescription management	Manages the closed-loop process of prescription issuance, verification, distribution, use, and refund of investigational product, and supports different blinding methods (such as open, single-blinding, and double-blinding), which can be traced
	Label management	All circulation links of investigational product support automatic label scanning and verification (including inbound, outbound, use, etc.)
	Intelligent detection	Intelligent early warning and verification of the management process of investigational product (such as near expiration date early warning, inventory early warning, and rule-based verification)
Financial management	Payment and allocation management	Manages the financial process of payment addition, allocation (such as subject fee, investigator fee, and inspection fee), review, etc
	Budget management	Manages the budget associated with the study project, including budget list, budget summary, and budget adjustment
	Expenditure management	Manages all kinds of expenses and related study project funds
	Workload statistics	Counts the corresponding workload in different dimensions (such as executive departments, project contracts, billing departments, and cost accounting) to produce financial statements
	Project funding amount	Manages the summary and detailed list of various expenses of study projects (such as subject fee, investigator fee, and inspection fee)
Quality management	Quality control data reporting	Manages the reporting of various quality control data (such as mid-term study quality control, study monitoring, study audit, and site audit)
	Quality control element capture	Automatically obtains corresponding quality control elements during clinical trial running (such as deviation protocol, out of visit window, and adverse events)
	Operational data query	With authorization, the quality controller can query the running data of the study project in real-time

The third level is backstage management, which is indispensable for the stable operation of the CTMS. Table 4 shows the categories and features for backstage management of clinical trials. In this level, the proposed system provides services to users through the PMMS. Unified management of all user information and data access permissions of the system through user and permission management provides a basis for the realization of the collaboration of users with different roles. Log management identifies the problems in the system operation and records all the data changes for verification. These two points are also an indispensable part of achieving secure data access. Data statistics are recorded according to the requirements of CTI to provide managers with statistical charts of study projects and operational data to provide decision support capability.

**Table 4:** Categories and features for backstage management of clinical trials

Category	Features	Description
User and permission management	User registration	Non-hospital users apply for registration and approval mode (such as clinical research coordinator (CRC) and clinical research associate (CRA))
	User list	User list contains all the users in the system, managing login policies and permissions
	Permission management	Realizes the role-based authority management system
Log management	System log	Includes system operation and data change logs, traceable to the source of changes
Data dictionary management	Basic data maintenance	Basic data support for the operation of the clinical trial management system (CTMS) can be maintained through a graphical interface
	Basic data synchronization	Sets up synchronization task; part of the basic data is automatically obtained from other business systems by means of timing synchronization
Data statistics	Data statistics	Statistical analysis capabilities of various data, support tables, and graphs
System settings	System settings	System customization functions to enable the system to operate more intelligently (such as messages and templates)

### Entire process management for clinical trial

**Fig. 2:**
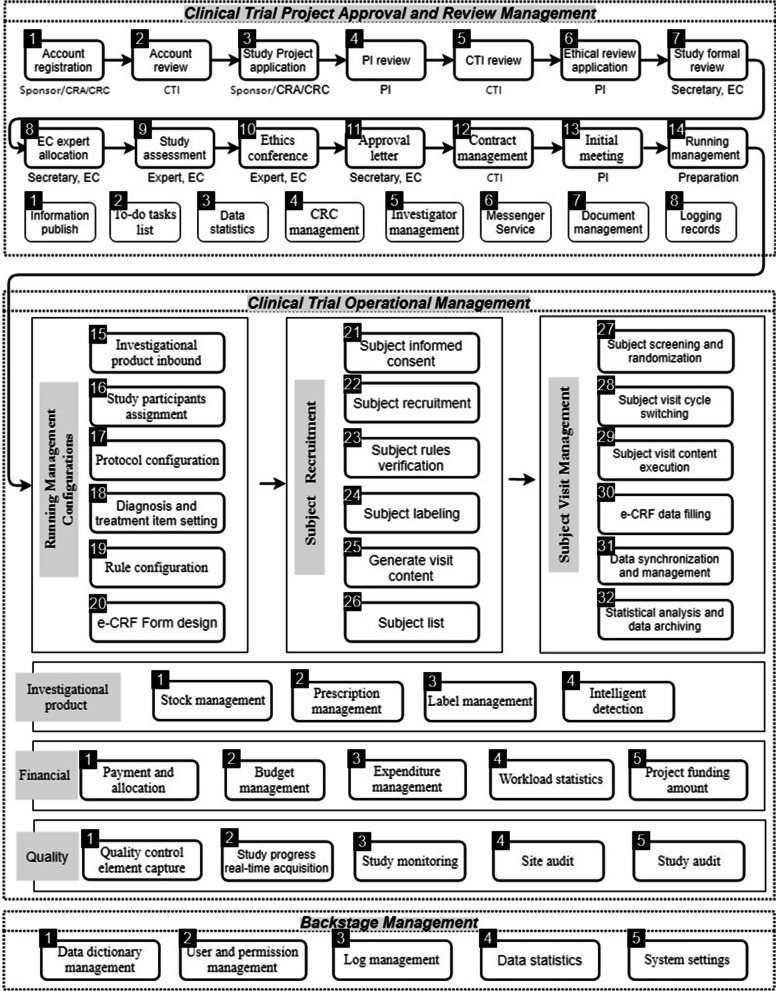
Process management system further categorized into 59 internal processes by combining user and authority management. CRA: clinical research associate; CRC: clinical research coordinator; CTI: clinical trial institutes; PI: principal investigator; EC: ethics committee

FAHZU CTMS realizes the entire process management of clinical trials through the organic combination of project approval and reviews management, clinical trial operational management, and backstage entire process management, which is further categorized into 59 internal processes by combining user and authority management (Fig. 2). Clinical trial project approval and review management consists of 14 main processes and eight auxiliary processes, which can efficiently complete the review and contract signature phases of the study project; a kick-off meeting is held to allow entry into clinical trial operational management. Clinical trial operational management is a complex and long-term process involving multiple factors such as subjects, diagnosis and treatment, medicine, and finance. We divide it into six stages: operational management pre-configuration, subject recruitment, subject visit management, investigational product management, financial management, and quality management, and then further subdivide these stages into 32 internal processes. Finally, backstage management, as the basic component, supports the stable operation of the entire clinical trial process.

### Data integration with external systems

**Fig. 3:**
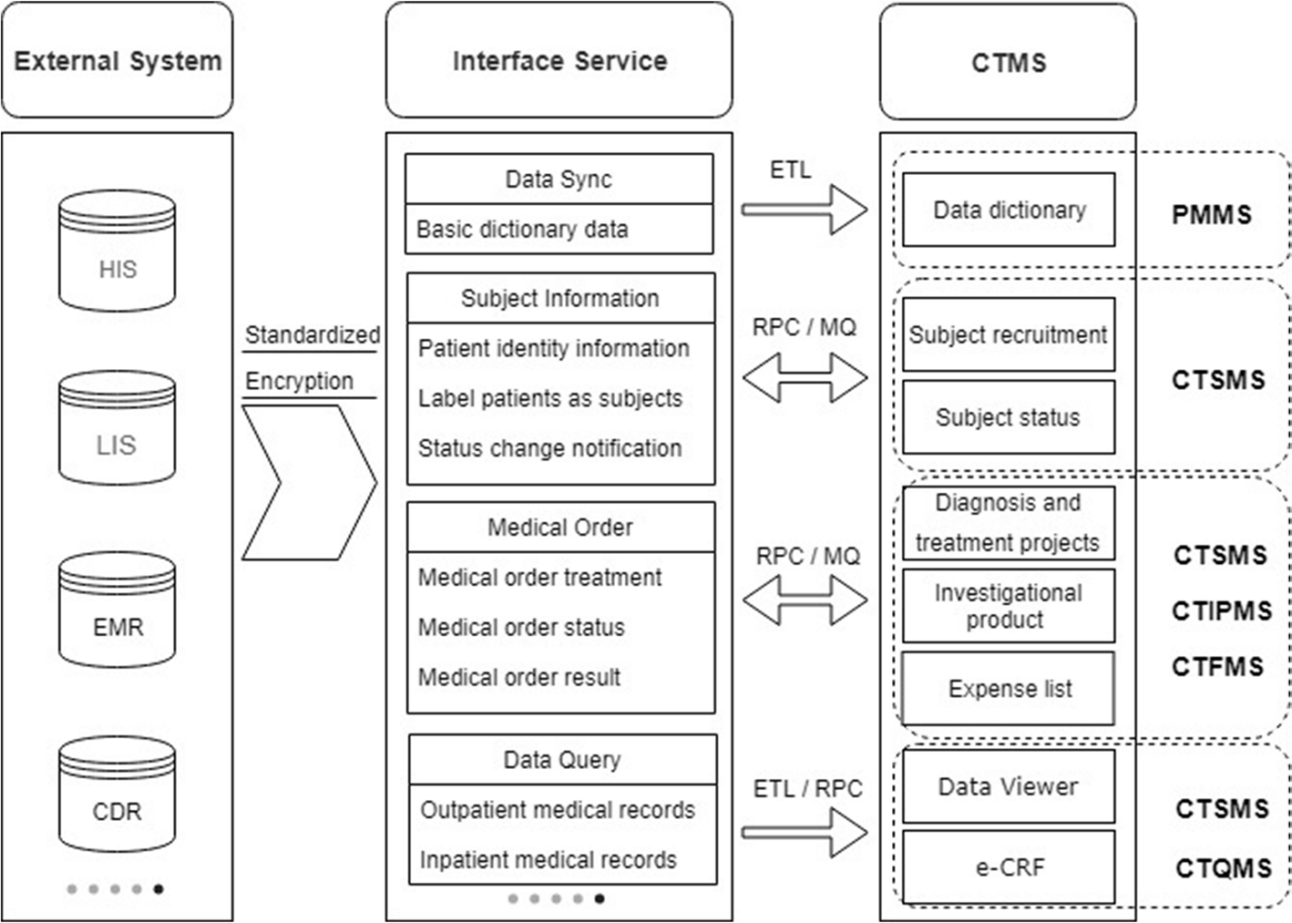
Critical services for data integration between the clinical trial management system (CTMS) and external systems. HIS: hospital information system; EMR: electronic medical record; LIS: laboratory information system; CDR: clinical data repository; ETL: extract-transform-load; MQ: message queue; RPC: remote procedure call; CTMS: clinical trial management system; PMMS: permission management and maintenance system; CTSMS: clinical trial subject management system; CTIPMS: clinical trial investigational product management system; CTFMS: clinical trial financial management system; CTSMS: clinical trial subject management system; CTQMS: clinical trial quality management system

To use the CTMS as a dedicated database for clinical trials, data integration with external systems is indispensable. Figure 3 shows some of the critical data integration services between the CTMS and external systems. CTMS subsystems integrate with external systems such as HIS, LIS, EMR, and CDR through the interface service platform to achieve data service standardization and transmission process encryption. The data integration method supports extract-transform-load (ETL), message queue (MQ), and remote procedure call (RPC) services and is flexibly selected based on the data volume and business model. Data synchronization and data queries involve a large amount of data. The operations use ETL batch extraction and RPC service real-time queries, which is used for data dictionary maintenance as well as queries of subjects’ full diagnosis and treatment data, and e-CRF structured data filling. Subject information data and medical order data use the synchronous call of RPC service and asynchronous notification of MQ. Synchronous calling is used to obtain the patient information of the subject recruitment, the subject’s diagnosis and treatment items, and prescription issuance, billing, etc. Asynchronous calling is used for notification of changes in the subject’s status, obtaining medical order status, obtaining results, etc.

### Data security and privacy protection

Data security and privacy protection ensure that a mature process is developed and maintained throughout the design life cycle. Data security primarily involves data storage and data access security. Privacy protection focuses on ensuring data security while complying with laws and regulations to de-identify sensitive patient information.

Data security mainly includes the two aspects of data storage security and data access security. Interms of data storage security, FAHZU CTMS adopts two strategies: (1) multi-node storage of data to achieve high availability of data services and a data backup strategy to ensure that data will not be lost in case of failure, and (2) encrypted storage of sensitive data (such as passwords, ID numbers, and bank card numbers). Policies for data access security are (1) security at the network level, through safety equipment (such as firewall, the host security protection, bastion host, and database/log audit tools), to implement access control and prevent illegal access, (2) security at the application level through multiple roles of authority management system access controls on data content, especially including rules to ensure no open access to prescriptions (such as CRC non-prescription issuance authority) as well as finding and repairing the vulnerabilities of the application through penetration testing and completing the information security technology-evaluation requirement for classified protection of cybersecurity—Level 3, and (3) maintaining a detailed data change log can be useful in tracing the record of the process of data recovery.

Subject privacy protection aims to protect sensitive patient information through a series of de-identification processes, which are explicitly required in the GCP. The FAHZU CTMS strictly implements the requirements of subject privacy protection in the GCP guidelines and refers to the Information Security Technology Personal Information Security Specification issued by the Standardization Administration of China and the HIPAA Regulations issued by the U.S. Department of Health. Final compliance with HIPAA’s Protected Health Information Disidentifiability Requirements Implementation Specifications excludes 18 personal health identifiers (PHIs) from the CTMS (such as name, address, cell phone number, and social security number) to comply with international and national laws. For CTMS users to identify subjects, we used the clinical trial protocol number, subject screening number, and subject name initials to uniquely identify them. At the level of data interaction, the patients’ medical record numbers in the hospital are systematically encrypted and stored in the CTMS database, which is linked to the data of external business systems (HIS, LIS, EMR, CDR, etc.) for automatic data transmission.

### Evaluation

The evaluation of the CTMS is ongoing and mainly conducted at two levels: clinical trial project approval and review management, and clinical trial operational management. The former focuses on the convenience of multi-party collaboration and the efficiency of project approval, whereas the latter focuses on the evaluation of subject-centered data management throughout the process and the improvement of the quality of clinical trials.

The improved clinical trial project approval and review management after applying the FAHZU CTMS, compared with that before the system was implemented, mainly embodies the following advantages: (1) It realizes process automation management based on task and combined with workflow technology, cooperates with user authority management systems, realizes automatic triggering of node events, and performs automatic assignment of processing personnel and message notifications such that personnel only need to process their personal to-do lists according to the message reminder, reducing the complexity of system use under multi-party collaboration. (2) Clinical trial project approval supports remote application, electronic document management enables online submission of project approval materials as well as online review and summary of revision opinions in the review process, which reduces the workload of project approval materials review and improves the efficiency of project review. Since CTMS began to operate in the hospital in December 2014, 1,388 study projects have been accepted and 43,051 documents have been submitted through CTMS as of March 31, 2021.

The focus at the clinical trial operational management level is centered on patient outcomes to achieve full-cycle data management. Here, the advantages are more significant. The core advantages are summarized as follows: (1) The CTMS requires that the research plan must be entered before the recruitment of subjects, and the visit content of the current visit cycle can be automatically correlated during the subject visit. It is transformed into to-do tasks in the current research stage, with timely reminders, reducing deviations from the protocol. (2) An independent billing model is adopted for clinical trial-related inspection and treatment expenses so that subjects can be exempted from expense reimbursement and be marked in the hospital business system to meet non-clinical trial diagnosis and treatment reminders and clinical trial inspection green special requirements such as channels. (3) The CTMS contains the data on each subject, including newly generated data during operation and data collected in the hospital business system. Through strict authority classification, the scope of the data queries, such as those issued by the CRC and other data query authorities, are limited to subjects who are responsible for the project. (4) Clinical trial drugs are label-based full-cycle closed-loop managed, and transfers are completed through label scanning, and support some special properties of clinical trial drugs, such as open, single-blind, and double-blind studies, and situations with other prescriptions, where drugs need to be random, and when the serial numbers and medicine need to be recalled.

**Fig. 4:**
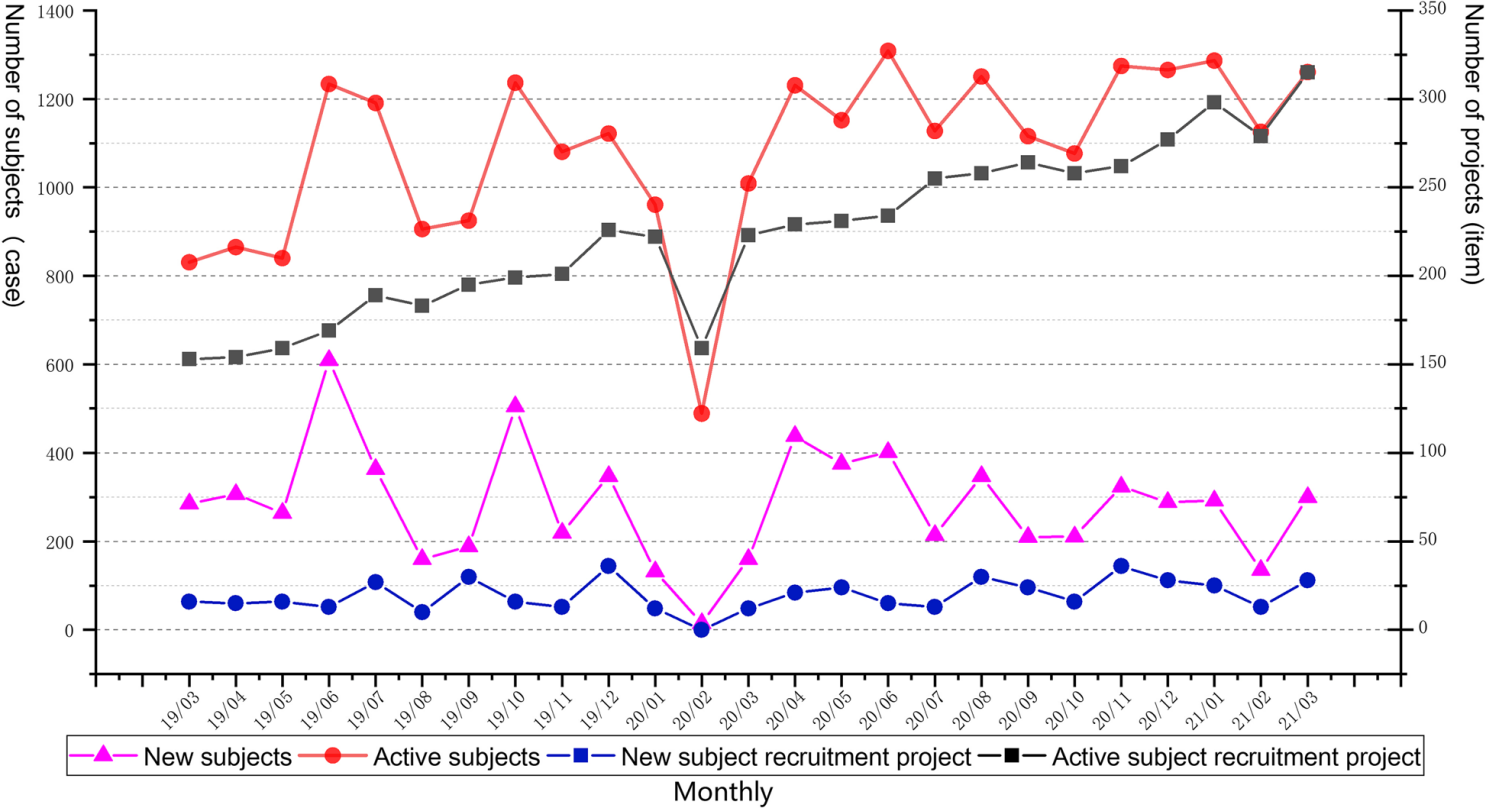
Change in the number of subjects and corresponding study projects enrolled in the clinical trial management system (CTMS) over the last two years

The clinical trial operational management of CTMS began a pilot run at FAHZU in March 2015. Pilot runs are conducted to validate and improve the effectiveness of system functions and compatibility with different types of clinical trials. Since September 2015, all newly initiated clinical trials on the site have been managed through CTMS. According to statistics, as of March 31, 2021, a total of 12,144 subjects have been included in the management of CTMS, across 472 study projects. Figure 4 shows the change in the number of subjects and corresponding study projects enrolled in CTMS in the last two years. We observed that the monthly number of new subjects and the corresponding study projects remained stable, and the number of active subjects also remained stable, while the corresponding monthly number of active projects increased. Since the outbreak of COVID-19 in China in December 2019, with the support of the integrated CTMS and necessary measures (such as remote follow-up, express delivery, and remote inspection), it may be observed from Fig. 4 that the clinical trials have continued at a steady rate during the COVID-19 outbreak.

## Discussion

Informatization is an inevitable trend in clinical trial management [9]. At present, there are many clinical trial informatization management systems in China; however, there are few systems that can connect with the information system of the clinical research center and unify the sponsor, data management department, and other parties into a platform for cooperation. The FAHZU CTMS has been researched and developed fully independently based on a distributed service architecture. It takes process management and trial data as the core, highly integrates, interconnects, and interfuses with hospital clinical business systems, and combines key contents such as data security and privacy protection to achieve independent application layers and interconnected data layers. Thus, comprehensive process management and dynamic real-time monitoring of clinical trials can be realized. Compared with commercial products, our self-developed CTMS is more cost-effective and highly customizable, fully fits the management needs of the hospital as a clinical trial administrative institution, and allows more timely system version updates, system operation, and maintenance response. Based on the deep understanding of hospital clinical business systems such as HIS, LIS, CDR, PACS, and EMR by information technology experts, a more comprehensive scheme was designed to achieve a high degree of integration between the CTMS and clinical business systems. The large amounts of medical data required for clinical trials are docked into the developed system and used as a CTMS-independent data collection and storage subsystem. In addition, based on the full understanding of data security and privacy protection, the security of the system was assigned great importance from the beginning of system development, and the privacy protection function of subjects was improved to better serve the entire process management of clinical trials in our institution. From an operational point of view, the use of the system has significantly improved the efficiency of researchers and institutional managers. For the institutional management, the system realizes real-time and efficient management of the entire process of clinical trials and ensures the reliability, authenticity, and integrity of clinical trial results. To ensure the safety of subjects, it realizes limited sharing of information through the data query, statistics, and tracking module. Institutional managers can view the project schedule and browse project-related node information, to accurately grasp the implementation of the project status, improve the quality of clinical trial data, reduce time consumption, and promote the standardized implementation of clinical trials according to the GCP guidelines and SOP of our hospital. On this basis, a full-cycle data quality monitoring and early warning platform for clinical trials can be constructed. Through artificial intelligence technologies such as text mining, natural language processing, machine learning, and knowledge mapping, intelligent full-cycle data analysis and early warning in clinical trials can be realized. These include the matching of inclusion and exclusion criteria, warning of inspection, warning of combined drug prohibition, warning of absence of visit activity or out of window, and intelligent warning of underreporting of AE or SAE.

The protection of private information is a more important consideration than the system construction [10–12]. The biggest problem with the establishment of the system is that the subject information may be exposed, which is a serious ethical problem. To solve potential risks from the level of laws and regulations, it is necessary to think deeply. Therefore, at the beginning of the design, we paid special attention to the concept of network data security and privacy protection, carried out the privacy impact assessment, and integrated the measures of privacy protection into the entire process of information system development. Institutional managers, researchers, quality controllers, CRCs, drug administrators, and inspectors are divided into different users. There is a strict permission management system for user accounts, to avoid the problem of account borrowing, certificate authority authentication or face recognition systems should be added to adopt fine-grained permission management. In addition, the SOP for risk assessment is particularly important, including the definition, classification, and rating of risks [13]. Only information management systems that meet the requirements of risk assessment can be operated. In the information age, everything is connected, and information is a trend [14, 15]. In today's highly developed Internet technology, we need to think about and solve the problems of data security and personal privacy, as well as the specific procedures to improve the efficiency of clinical research. At the same time, compliance with the Chinese GCP and International Conference on Harmonisation (ICH) requirements for GCP ensure compliance with ethical and legal provisions.

Compared with the traditional paper CRF, e-CRF allows researchers to enter the CRF electronically based on the source data rather than fill it in manually [12]. However, source data verification (SDV) is still required to compare the CRF with original medical records and inspection lists to ensure the quality of data input. Our CTMS is now able to automate the collection of structured data, eliminate the SDV process, and further simplify the process. Thus, any modifications to the electronic source data can be recorded through an audit trail. In future, our system will also study the complex natural language analysis involved in unstructured data. Simultaneously, we will build a way to connect electronic data acquisition (EDC) and hospital e-CRF to directly collect and transfer clinical data electronically, ensuring the quality and integrity of the data.

In future, we also want to utilize the data in the clinical trial management platform to realize intelligent subject recruitment and improve the efficiency of subject recruitment. Recruiting subjects for some clinical trials is difficult, especially those involving rare diseases, stringent admission criteria, and special subgroups. Owing to information asymmetry, researchers only know the condition of the patients they are treating, and patients do not know that their disease may be under research at the hospital, which can seriously affect the progress of clinical trials [16–19]. To fully utilize the hospital CTMS system of clinical data, the central hospital and administering medical hospital can simultaneously be on the same clinical trial management platform, which will further enrich the patient resources. Additionally, the system can provide search functions, select exclusion criteria, fast-matching potential subjects, and realize intelligent recruitment of subjects, which can effectively help the sponsor accelerate the clinical trial process, reduce R&D costs, and successfully seize market opportunities. However, in the era of big data, these data have important scientific value in the field of clinical research. Applications such as large data analysis found that local residents’ disease condition, and its influencing factors—specific studies on key diseases—have been successfully applied to determine the time of disease distribution, location distribution, population distribution, and analysis of risk factors of disease; furthermore, it has been used for the evaluation of the effectiveness of clinical screening and diagnosis methods, inspection treatment or drug treatment effect, optimization of individual diagnosis and treatment of disease, and research on the influencing factors of diseases after intervention. This is to provide the basis for the local health administration departments to make health management decisions.

Since the outbreak of COVID-19, many jobs around the world have been stalled to a certain extent. In the event of a major public health emergency, carrying out clinical trials and ensuring the smooth implementation of monitoring work has become a problem for clinical trial practitioners, and remote monitoring has therefore been put on the agenda. The U.S. Food and Drug Administration has long encouraged more centralized monitoring, where inspectors perform inspections in the office using relevant information tools rather than at research institutions (hospitals). The development trend of clinical trial monitoring is to replace on-site monitoring with centralized monitoring [20]. Remote monitoring can improve the quality and efficiency of clinical research and reduce its cost, and it is possible only when a series of electronic clinical trial products such as CTMS, electronic data acquisition, EMR, and clinical data management systems are widely used. The clinical trial information management system of our hospital is a CTMS that unites multiple teams on one platform and is highly integrated with all clinical systems of the hospital. Based on the Internet platform, remote data monitoring and cloud auditing can be based on the CTMS as a mature operation.

Compared with other information systems, CTMSs are more professional and personalized. The realization of the effectiveness of a CTMS requires a significant amount of time, and the more clinical trials undertaken, the more significant the effect. Compared with the system design, the comprehensive and efficient application of the system takes longer to achieve. With the digitization of clinical research, the sharing and integration of research data will bring many management advantages, such as an increase in available management resources, scientific and data support for major decisions, the convenience of remote management, pertinacity, and pre-operation. The CTMS developed by our hospital will be constantly updated and upgraded, and its functions will constantly improve. The system update will keep pace with developments in international drug clinical trial management, promote the development of clinical trials in a more standardized direction, and promote the disciplinary status of the hospital regarding high-level clinical trials.

## Conclusion

The FAHZU CTMS, as the first integrated CTMS independently developed by a hospital in China, can better adapt to the institutional needs for individualized, whole-process, and dynamically comprehensive evaluation and supervision of clinical trials. The integrated CTMS contains three levels and seven subsystems, which fully realizes the whole-process data management of clinical trials from project approval and review management to operational management. Through the unified interface system, the developed CTMS provides a variety of access methods to complete efficient data integration with the clinical business systems, and applies multiple security policies combined with privacy protection methods to effectively ensure the security of data and the privacy of subjects during clinical trial operation. The operation results based on the integrated CTMS show that it can effectively control the risks in the clinical trial process, so as to improve the science, safety, and timeliness of the new drug development process.

## Data Availability

FAHZU CTMS consists of several subsystems. Considering data security and privacy protection, subsystems associated with subject data are deployed based on the hospital’s internal network, and domain names are resolved by a self-built domain name systemserver. The following four links show part of the web pages of the core subsystem, which can show the function design and data volume of the system. For additional information please contact the corresponding author. 1. https://doi.org/10.5281/zenodo.5880663 2. https://doi.org/10.5281/zenodo.5880783 3. https://doi.org/10.5281/zenodo.5880799 4. https://doi.org/10.5281/zenodo.5880826

## Abbreviations

AE: Adverse event
CDISC: Clinical Data Interchange Standards Consortium
CDR: Clinical data repository
CRA: Clinical research associate
CRC: Clinical research coordinator
CTEMS: Clinical trial ethical management system
CTFMS: Clinical trial financial management system
CTI: Clinical trial institution
CTIPMS: Clinical trial investigational product management system
CTMS: Clinical trial management system
CTPMS: Clinical trial project management system
CTQMS: Clinical trial quality management system
CTSMS: Clinical trial subject management system
e-CRF: Electronic case report form
EC: Ethics committee
EMR: Electronic medical record
ETL: Extract-transform-load
FAHZU: First Affiliated Hospital, Zhejiang University School of Medicine
GCP: Good Clinical Practice
HIPAA: Health Insurance Portability and Accountability Act
HIS: Hospital information system
HTTPS: Hypertext transfer protocol secure
ICH: International Conference on Harmonisation
LAN: Local area network
LIS: Laboratory information system
PACS: Picture archiving and communication systems
PHI: Personal health identifier
PI: Principal investigator
PMMS: Permission management and maintenance system
R&D: Research and development
RPC: Remote procedure call
SAE: Serious adverse events
SDV: Source data verification
SSO: Single sign-on
UI: User Interface
UX: User Experience
